# Knowledge on Prevention and Management of Preeclampsia and Eclampsia among Nurses in Primary Health Settings: Baseline Findings from an Interventional Study in Dodoma Region, Tanzania.

**DOI:** 10.24248/eahrj.v4i1.619

**Published:** 2020-06-26

**Authors:** Joho A Angelina, Stephen M Kibusi, Ipyana Mwampagatwa, Alex Ernest

**Affiliations:** a Department of Nursing and Midwifery, College of Health Sciences, University of Dodoma; b Department of Public Health, College of Health Sciences, University of Dodoma; c Department of Clinical Medicine, College of Health Sciences, University of Dodoma

## Abstract

**Background:**

Preeclampsia and eclampsia are conditions which increase maternal and foetal morbidity and mortality worldwide. These conditions are ranked as the second leading cause of maternal deaths. Nurses have a critical role in preventing and managing preeclampsia. However, their knowledge has not been evaluated particularly among those working in primary health facilities, where opportunities for continue education is limited.

**Objective:**

To assess knowledge on prevention and management of preeclampsia and eclampsia among nurses working in the primary health care settings.

**Methods:**

Analysis of baseline data from an intervention study which test the effectiveness of simulation-based training on obstetric and neonatal emergencies among nurses in managing maternal and newborn emergencies in primary health care settings. A total of 39 primary health centres within 7 districts in Dodoma Region were selected to take part in the interventional study. Individual participants were nurses working in maternity units were involved. 172 nurses were selected using a simple random method. Nurses' knowledge on prevention and management of PEE and its predictors were assessed using a self-administered questionnaire. Descriptive statistics analysis was done to determine the distribution of the background characteristics of nurses and logistic regression analysis was performed to explore predictors of nurses' knowledge

**Results:**

Overall knowledge on preeclampsia and eclampsia was 88 (51.2%). Professional qualification was a predictor associated with a nurse's knowledge about preeclampsia and eclampsia. Registered nurses were more knowledgeable compared to enrolled nurses (AOR3.311; CI, 1.62 to 6.768; *P* value =.001), years of working experience showed no association with knowledge on preeclampsia and eclampsia (AOR 0.98; CI: 0.39-2.47; P values = 0.970)

**Conclusion:**

This study showed there is a critical knowledge deficiency in the prevention and management of preeclampsia and eclampsia among nurses working in maternal units of primary health care setting. Effective regular training on prevention and management of preeclampsia and eclampsia for frontline nurses is required in order to improve maternal and neonatal survival.

## INTRODUCTION

Worldwide, maternal and neonatal deaths are still a challenge and it is estimated to be 211 maternal mortality rates per 100,000 livebirths as of 2017. This represents a decrease of 38% from 2000^1^. However, neonatal mortality was estimated to be 18per 1000 livebirth as of 2017. This represents a reduction of 30.6% from 2000.^2^ In Sub-Sahan Africa, maternal mortality rate was estimated to be 525 per 100,000 livebirth as of 2017, a reduction of 38.7% from 2000^1^. Furthermore, the neonatal mortality is still high and estimated to be 27.2 per 1000 live birth as of 2017, this is a reduction of 40.7% from 2000.^2^ In Tanzania, there is no significant change in maternal and neo natal deaths, it is estimated that maternal mortality rate (MMR), has reduced from 854 as of 2000 to 524 deaths per 100,000 livebirths in 2017^1^. While neonatal mortality rate (NMR) was 26 in 2010, it was 25 deaths per 1,000 livebirths as of 2015^3^. Hypertensive disorders in pregnancy are responsible for about 26% of maternal deaths worldwide, in Africa the disorders cause deaths for about 99%^4^. The vast majority of maternal deaths occur in low income countries. The Tanzania Demographic and Health Survey (TDHS) 2015-16 reported that sixteen percent of maternal deaths were due to hypertensive disorders, including eclampsia^5^. Furthermore, PEE are the main cause of maternal, foetal and neonatal mortality especially in low resource countries^5^. Preeclampsia and eclampsia (PEE) are serious conditions which increase long term disability, maternal and foetal, morbidity and mortality worldwide.^6,7^ Common hypertensive conditions during pregnancy include: new onset of high blood pressure during pregnancy (gestational hypertension), chronic hypertension, preeclampsia and eclampsia. Signs and symptoms of preeclampsia include: systolic blood pressure >140 mmHg, diastolic blood pressure above 90 mmHg, proteinuria (above 0.3g/24h), frontal headache, visual disturbance and epigastric pain and substantial maternal organ dysfunction^8^. Complications of eclampsia include cardiovascular disease, renal disease, cerebrovascular disease and shorten life expectancy^9^. Additionally, adverse foetal effects from PEE include intrauterine growth restriction, small for gestational age, respiratory distress syndrome, transient tachypnea of the newborn, anaemia, apnea, asphyxia, perior intraventricular haemorrhage, cardiomyopathy, cerebral palsy^10,11^ and persistent pulmonary hypertension of the newborn^12^. Furthermore, preeclampsia and eclampsia are leading causes of perinatal mortality^11^.

There are complex factors that impact timely care of women with PEE. Lack of competent frontline (nurses) health care providers' diagnosis and management especially in the area of maternal and neonatal emergency care may account for these deaths^13^. This account for third delay model for appropriate and timely management. However, if the woman comes to the ante-natal clinic (ANC) or labour ward early to receive appropriate care while the nurse receiving the woman does not know how to diagnose and manage the PEE, this would put the woman and her unborn baby at increased risk of severe morbidity and or deaths.

Early detection, rapid response, accurate management and timely delivery of women with preeclampsia with severe features and eclampsia reduces maternal and foetal complications and deaths. Nurses' knowledge and skills about diagnosis and management of these conditions is critical factor in maternal and neonatal morbidity and mortality.

In Tanzania, the primary health centres are the entry point for most patients including pregnant women who seek health care services whenever needed. The primary health care centres are run by the clinical officers and few by the medical officers. Furthermore, majority of nurses who are working in the health centres are those with certificate and few with diploma in nursing. Moreover, the nursing training for those with diploma covers maternal and neonatal emergencies including PEE while those with certificate are not trained in maternal and neonatal emergencies^14^. Nurses with certificate level of training are able to conduct normal delivery only, and equipped with skills to assist their senior nurses with diploma or health care providers (clinical and medical officers) in emergency or complicated labour. However, in real situation these nurses in some of health care centres are working independently without guidance from higher level nurses, clinical or medical officers.

In the rural areas most of the health centres are public ones, while in urban there are public and private health centres. In Dodoma, the health centres have the capacity of serving an average of 10,772 population, and the number of nurses per health centre ranges from 3 to 20.

The referral health system in Tanzania is of a pyramidal pattern operating upwards from the lowest level which is the community. Patients are referred from community to dispensary, then to health centres, to district, to regional referral hospital, to consultant hospitals and national and or specialized hospitals. The referral system of patients from one level to another follows the skills which are required to address the problems of the patients. Moreover, the government has established an open-door policy, and hence patients can be referred from lower level to higher if the other referral levels lack the required skills^15^.

Nurses are crucial frontline healthcare providers in Tanzania's healthcare facilities. Their knowledge and skills in managing obstetric emergencies are of paramount importance. They must be able to provide timely lifesaving emergency care and correctly identify women needing referral to a higher level of care^16^. Healthcare training programs for nurses on the prevention and management of eclampsia have been shown to reduce adverse outcomes in critical patientcare settings^17^. Moreover, strengthening on the job training regarding emergency care helps to improve nurses' level of knowledge of PEE management^18^.

Essential knowledge for handling maternal emergencies, including eclampsia, is a prerequisite to appropriate treatment and referrals for mothers. Lack of core knowledge leads to poor decision making and management of emergency maternal health conditions. Furthermore, insufficient knowledge can be attributed to delays in treatment and referrals^19,20^. Adequate knowledge is required to correctly identify women with preeclampsia with and without severe features. This foundational knowledge helps ensure timely evidence based decision making and care based on international guidelines. In Central Tanzania, the knowledge of nurses about the management of PEE, is not well known. Therefore, the aim of this study was to assess nurses' knowledge on managing PEE and identify the predictors of nurses' knowledge in the primary health care setting in Dodoma Region.

## METHODS

### Study Design and Target Population

This paper present analysis of baseline data from an intervention study which aim at testing the effectiveness of simulation-based training on obstetric and neonatal emergencies among nurses in managing maternal and newborn emergencies in primary health care settings. The study was conducted between May and June 2017, exploring nurses' knowledge on PEE. Study participants were nurses working in antenatal ward, labour, postnatal and theatre units.

### Study Setting

This study was conducted in all seven districts in Dodoma Region namely Mpwapwa, Kogwa, Chamwino, Dodoma Municipal, Bahi, Chemba and Kondoa. A total of 39 health centres in the region (27 government, 4 faith based organization, 3 parastatal and 5 private). All health centres included in the study are providing ANC services free of charge. On average, a health centre serves 10,772 population, and staffed with 3 to 20 number of nurses. Dodoma Region was selected for this study because of high maternal deaths of 512 per 100,000 livebirths compared to the surrounding regions Singida (468), Manyara (376) and Iringa (292) per 100,000^21^.

### Sampling Technique and Sample Size

The districts were selected purposively due to high maternal deaths in Dodoma Region and all 39 health centers in Dodoma were included in the study. Simple random method was used to select participants from maternity units (antenatal, labour ward, and postnatal) and theatre unit. A 50% rule based on researcher's convenience was considered as a criterion for a decision for the number of nurses to recruit from health centres. Therefore, for each health centre half of its nurses were included in the study. This is due to shortage of nurses, in the health centres, nurses are working in multiple sections, and they don't have specific work. A self-administered questionnaire was used to assess nurses' knowledge on the prevention and management of PEE. A total of 176 study participants were recruited based on a sample size calculation using the Kish and Leslie formula^22^ (n = Z^2^ p(1-p)/**e^2^**) whereby n is a minimum sample size, z is a constant, standard normal variation (1.96 for 95% confidence level) p =11.8% a prevalence reported by the study conducted in Moshi municipality in northern Tanzania on the evaluation of knowledge and management practices of hyper tension in pregnancy^23^. Considering a margin of error (e) of 5%. The minimum required sample size was 160 and we added 10% for non-response. The total sample size was 176 nurses and response rate was 97.7%.

### Data Collection Tool

In this study, data was collected using a standardized questionnaire in Kiswahili, the national language of Tanzania. Knowledge about PEE was assessed using a standardised and validated questionnaire which contained 10 items with a total of 20 correct answers. The tool was adapted from Jhpiego education materials^24,25^. The questionnaire contained three sections. The first section reviewed the nurses' demographic characteristics, the second section explored their educational background and the third section assessed nurses' knowledge about the management of obstetric emergency including PEE. Each item with a correct response received one point and incorrect responses were scored as zero. Respondents who scored 15 marks and above (75% and beyond) were categorized into adequate knowledge group. Those who scored below 15 marks were categorized into inadequate knowledge group. Before the actual data collection process, a pilot study was conducted with 20 nurses working at district hospital to test the tool's ability to obtain needed information prior to data collection and to identify confusing or ambiguous questions. Ambiguous questions were reworked or removed. Nurses included in the pilot study were not included in the final study.

### Data Collection Procedure

The study was conducted in the antenatal ward, labour ward, postnatal ward and theatre units where nurses provide care to mothers with PEE. Participation in the study was voluntary. Self-administered questionnaire was completed by each nurse in the presence of researcher and assistant. Clarification was provided in case participants were not clear or had questions about the tool.

### Dependent and Independent Variables

The dependent variable was the knowledge of nurses on management of PEE. Independent variables included age, sex, experience working in the maternal unit, professional qualification, duration of professional training and timing of work shift.

### Data Analysis

Data entry and statistical analysis were performed using IBM SPSS Statistics for Windows version 20.0 (IBM Corp, Armonk, NY, USA)^26^. Descriptive analysis was performed to explore distribution of demographic characteristics of respondents. Knowledge items were computed to obtain total knowledge score which was categorized into adequate knowledge and inadequate knowledge. Thereafter, a cross tabulation analysis was performed to assess relationship between categorical variables. Chi-squaretest was used to determine significant relationship between categorical variables. Significant relationships were further analyzed by performing simple logistic regression analysis. All variables with significant relationship with knowledge (*P* value < .05) were included in the multiple logistic regression.

### Ethical Considerations

Ethical clearance was obtained from the Institutional Research Review Committee of the University of Dodoma in Dodoma Region, Tanzania. Permission to conduct the study was obtained from the Regional Medical Officer and the District Medical Officers from respective districts within Dodoma Region. The aim of the study was clearly explained to the participants before signed the consent form. The participants were informed that participation in the study was completely voluntary and they can withdraw at any stage without incurring any consequences. There was no reasonable risk of harm to the participants. The anonymity of the participants was ensured by not having any identification on the data collection tool so that information would not be traced back to individuals. Confidentiality was guaranteed by storing data in a safe and locked place, and only the researcher had access to the raw data.

## RESULTS

A total of 172 nurses from 39 health centres in Dodoma Region participated in the study. This corresponds to a 97.7% response rate. Mean age the nurses who responded to the questionnaire was 37.3 years (SD±11.393) and majority were females 145 (84.3%) and most 109 (63.4%) being enrolled nurses (certificate level of training). Three quarter of them had completed educational training of less than three years with slightly above half of them working in maternity unit for less than 5 years and received training on management and prevention of PEE as part of their nursing education (See Table 1).

**TABLE 1.** Nurses' Demographic Characteristics N=172

Demographic characteristics		Frequency	Percentage (%)
Sex	Male	27	15.7
	Female	145	84.3
Age Group (Years)			
	20-29	63	36.6
	30-39	39	22.7
	40 and above	70	40.7
Level of professional			
	Enrolled nurses	109	63.4
	Registered nurses	63	36.6
Years of professional training			
	Less than or equal to 3	120	69.8
	Above 3	52	30.2
Experience in the health sector			
	Less than 5 years	70	40.7
	5 years and above	102	59.3
Experience in maternity (years)			
	Less than 5 years	95	55.2
	5 years and above	77	44.8
Pre-service training on eclampsia management			
	Yes	90	52.3
	No	82	47.7
Night shifts			
	No	56	32.6
	Yes	116	67.4
Number of shifts per day			
	Two	27	15.7
	Three	145	84.3

### 

#### Level of nurses' knowledge on management of pre-eclampsia and eclampsia

Based on the operational definition used in this study, only 88 (51.2%) were found to have adequate knowledge on the management of pre-eclampsia and eclampsia. Those who scored 75 percent and above on the self-administered questionnaire were categorised as having adequate knowledge (See Figure 2).

**FIGURE 1.**
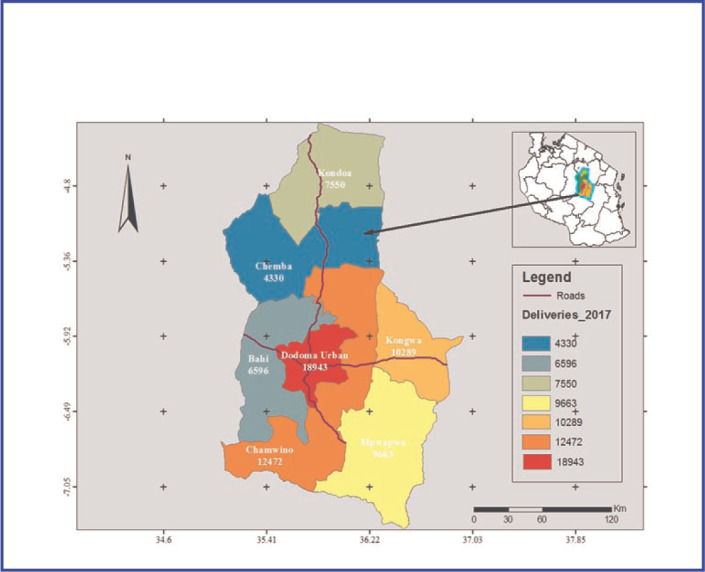
Distribution of number of deliveries per District in Dodoma Region for the year 2017

**FIGURE 2.**
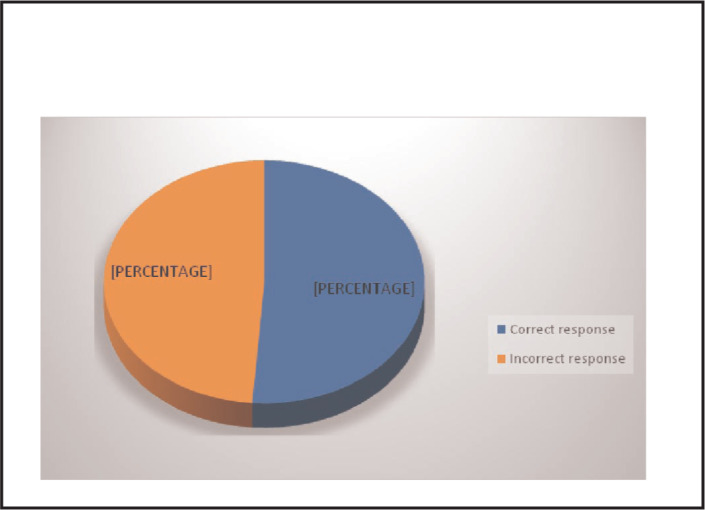
Knowledge Level Regarding Pre-eclampsia and Eclampsia Management

#### Nurses' responses to question related to knowledge on prevention and management of eclampsia

Nurses were given 10 multiple answer questions, with total of 20 correct answers. Most nurses 160 (93%) were able to correctly identify the recommended drug to be used for prevention and management of severe preeclampsia and eclampsia and 148 (86%) nurses were able to recognize the drug of choice for the management of high blood pressure. However, few nurses, 67 (39%), were able to know the type of intravenous fluid to be given to mothers with eclampsia (See Table 2).

**TABLE 2.** Number and Percentage of Nurses with Correct Responses to Prevention and Management of Preeclampsia and Eclampsia

Questions	Frequency (%)
**What are the immediate managements during fit?**	
Shout for help	112(65.1)
Ensure the woman airway is open	143(83.1)
**What care should be provided for a women after convulsion?**	
If available give oxygen 4-6 liters per minutes by mask or canula.	124(72.1)
Observe color for cyanosis and need for oxygen.	82(47.7)
Aspirate the mouth and throat as necessary	155(90.0)
**What kind of assessment/physical examination needed after convulsions/fits**	
Observe color for cyanosis and need for oxygen, Check for aspiration: lungs should always ascultated after the convulsion has ended, Check vital signs and fetal heart rate.	87(50.6)
**The recommended intravenous line for managing eclampsia is**	
Normal saline (NS) or Ringer's lactate	67(39.0)
**The recommended drug used to control convulsion in management of eclampsia is Magnesium sulfate**	160(93.0)
The recommended dose of that drug (selected in question 5) during control of convulsion is Gives Magnesium sulfate 20% solution, 4g IV slowly over 5-10 minutes OR 10 g of Magnesium sulphate, each buttock 5g with 50% solution deep IM injection with 1 ml of 2% lignocaine in the same syringe.	66(38.4)
**What is the prevention of toxicity of drug selected in question 5?**	
Assess respiratory rate. Is respiratory rate at least 16 per minute?	131(76.2)
Assess patellar reflexes. Is Patellar reflexes present?	113(65.7)
Assess urinary output. Is Urinary output at least 30 mL per hour over preceding four hours?	133(77.3)
**What are the immediate measures in case the toxicity of the drug selected in question 5 happens?**	
Withhold or delay the drug if respiratory rate falls below 16 per minute	86(50.0)
Withhold or delay the drug if Patellar reflexes are absent and Urinary output falls below 30 ml per hour over the preceding 4 hours.	94(54.7)
Assess ventilation and give Calcium Gluconate 1gm (10 ml in 10% solution).	127(73.8)
If diastolic blood pressure remains above 110mmhg, the recommended group of drugs used is Antihypertensive drugs (nifedipine or hydralazine)	148(86.0)
**Others management of eclampsia includes**	
Insert an indwelling urinary catheter to monitor urinary output and proteinuria, do a bed side clotting test, never leave the woman alone (convulsion followed by aspiration of vomit may cause death to a woman and fetus)	93(54.1)
Record drug administration and findings on the woman ‘s record, Share your findings to a woman and as appropriate to her partner or family member	91(52.9)
Explain management, based on diagnosis, and the importance for pregnancy, labor and delivery.	60(34.9)

This Table describe the steps of management of pre-eclampsia and eclampsia; showing nurses response to the given questions

#### Factors associated with nurses' level of knowledge on prevention and management of preeclampsia and eclampsia.

In cross tabulation analysis, nurses' level of knowledge on prevention and management of PEE was significantly associated with age (*P*<.001), professional qualification (*P* value =.002), duration of professional training (*P* value =.034), working experience in the health sector(*P*<.001) and experience in maternity unit (*P*<.001). High proportion of older (70%), more qualified nurses (66%) and those with longer years of professional training (64%) and experience (63%) had adequate knowledge on management of PEE (See Table 3).

**TABLE 3.** Factors Associated With Nurses' Level of Knowledge on Prevention and Management of Preeclampsia and Eclampsia (N=172)

Demographic characteristics		Knowledge on PEE management		x^2^	P-value
		Inadequate(%)	Adequate (%)		
**Sex**	Male	17(63.0)	10(37.0)	2.558a	0.110
	Female	67(46.2)	78(53.8)		
**Age (years)**					
	20-29	42(66.7)	21(33.3)	18.348a	0.001
	30-39	21(53.8)	18(46.2)		
	40 and above	21(30.0)	49(70.0)		
**Level of professional**					
	Enrolled nurses	63(57.8)	46(42.2)	9.564a	0.002
	Registered nurses	21(33.3)	42(66.7)		
**Duration of professional training**					
	Less than or equal to 3	65(54.2)	55(45.8)	4.512a	0.034
	Above 3	19(36.5)	33(63.5)		
**Experience in the health sector**					
	Less than 5 years	46(65.7)	24(34.3)	13.456a	0.001
	5 years and above	38(37.3)	64(62.7)		
**Experience in maternity unity**					
	Less than 5 years	57(60.0)	38(40.0)	10.583a	0.001
	5 years and above	27(35.1)	50(64.9)		
**Number of shifts per day**					
	Two shifts	10(37.0)	17(63.0)	1.785a	0.182
	Three shifts	74(51.0)	71(49.0)		
**Working night shift**					
	No	28(50.0)	28(50.0)	.045a	0.832
	Yes	56(48.3)	60(51.7)		
**Pre-service training on eclampsia**					
	Yes	49(54.4)	41(45.6)	2.375a	0.123
	No	35(42.7)	47(57.3)		

In binary logistic regression, age, professional qualification, years of training and work experience maintained their significant association with nurses' level of knowledge on management of PEE. Registered nurses, those aged 40 years and above, and those with longer duration of professional training and working experience were more likely to have adequate knowledge on management of PEE (Table 4). However, in multiple logistic regression, only professional qualification maintained its significant association with knowledge on management of PEE. Registered nurses were three times more likely (AOR 3.3; 95% CI, 1.6 to 6.8) to have adequate knowledge on management of PEE than enrolled nurses (See Table 4).

**TABLE 4.** Predictors of Nurses' Knowledge on Prevention and Management of Preeclampsia and Eclampsia (N =172)

Demographic characteristics	95% C.I.				95% C.I.			
	OR	Lower	Upper	p value	AOR	Lower	Upper	p value
**Sex**								
Male	1							
Female	1.98	0.85	4.62	0.114				
**Age (years)**								
20-29	1							
30-39	1.71	0.76	3.89	0.197	1.20	0.34	4.28	0.776
40 and above	4.67	2.25	9.70	0.000	4.19	0.97	18.22	0.056
**Professional qualification**								
Enrolled	1							
Registered	2.74	1.43	5.23	0.002	3.31	1.62	6.77	0.001
**Duration of professional training**								
< 3 years	1							
≥ 3 years	2.05	1.05	4.01	0.035	0.98	0.39	2.47	0.970
**Experience in H/S**								
<5 years	1							
≥ 5 years	3.23	1.71	6.10	0.000	1.35	0.35	5.25	0.661
**Experience in the maternity unit**								
< 5 years	1							
≥ 5 years	2.78	1.49	5.18	0.001	0.96	0.31	2.96	0.937

## DISCUSSION

This study aimed to assess nurse's knowledge of PEE, their prevention and management and associated factors among nurses working in the primary health care setting in Dodoma Region, Tanzania. Out of 172 study participants, 88 (51.2%) had adequate PPE management knowledge. Nurses' knowledge level on management of PEE in the current study was somehow comparable to a previous study conducted in public health facilities in Dar es Salaam, Tanzania which reported 76 (55%) of nurses as knowledgeable^27^ Nurses in the current study were less knowledgeable compared with results of the study conducted in Egypt which reported that 30% of nurses had poor knowledge regarding eclamptic care^28^. However, another study conducted in Egypt reported that 17% of nurses had optimal knowledge on management of preeclampsia^8^. Differences between these studies may be explained by approaches to pre-service and in-service training, lack of empowerment and also, lack of supportive supervision. Regardless, findings from all of these studies demonstrate that knowledge on PEE management is low among frontline nurses in these low resource settings.

Although majority of nurses (93.6%) knew that magnesium sulphate (MgSO4) is the drug of choice for the management of woman with eclampsia or preeclampsia with severe, few nurses (38.4%) knew its maintenance dose and how to correctly assess for toxicity after administration of MgSO4. Our study results are similar to other studies whereby nurses were also able to correctly identify MgSO4 as the medication of choice. However, the specifics of MgSO4 dosing, administration and monitoring is of grave concern^29^. The low knowledge observed in the current study for the management of PEE, may reflect low knowledge in other areas of maternal health care, including antenatal, intrapartum and postpatum care, which ultimately affects maternal and neonatal outcomes. Appropriate and timely care at the health facilitity is crucial to improve outcomes and avoid delays in care. Better understanding the factors that contribute to such delays will help the health system improve care quality and outcomes^20^. The current study revealed majority had been working as nurses in maternity for less than five years. This means nurses with good working experience in maternal and neonatal emergencies management are not retained in the labour ward. Our results differed with study conducted in Northern Nigeria whereby most of health providers had mean number of 9.6 year experience in managing PEE, and most of them knew MgSO4 is the drug of choice in preventing and treating PEE.^30^ The experienced nurses in labour ward should be considered as a good resource for teaching newly employed nurses on management of eclampsia and other maternal and neonatal emergencies.

The current study revealed educational preparation as a key predictor of an individual nurse's knowledge of management of preeclampsia and eclampsia. After controlling for possible confounders, registered nurses were three more likely to be knowledgeable about PEE management compared to enrolled nurses. In Tanzania, registered nurses complete educational training programs for 3 years (2008-present) or 4 years (prior to 2008) compared to enrolled nurses who receive 3 years (prior 2008) or 2 years (2008-present) of educational preparation. A difference in knowledge and also skills among differently trained nurses has been demonstrated in other settings in Sub-Saharan Africa^31^. Policymakers and educators need to consider the effectiveness of 2 year certificate nurse training programs compared with registered nursing training programs. It appears that there are major knowledge gaps despite comprehensive curricula. Innovative approaches are needed in all types of nurse training programs in Tanzania to ensure foundational knowledge and skills in obstetric and neonatal emergencies, including severe preeclampsia and eclampsia. To ensure that every birth attendant is truly skilled^32^, frontline nurses require core knowledge and fluency in lifesaving skills for mothers and neonates. To that end, there is a need to examine the duration of study for li-censure and effectiveness of pre-service training for nurses in Tanzania.

## CONCLUSION

This study demonstrated a critical gap in knowledge among frontline nurses providing maternal health care in Dodoma, Tanzania. One wonders about other key knowledge gaps among maternal health nurses in this setting. At a minimum, nurses are in dire need of regular refresher trainings that focus on lifesaving knowledge and skills for obstetrics, including the management of pre-eclampsia and eclampsia. Strengthening on job training will help nurses' readiness for obstetric emergencies, including early detection, proper management and timely referral. By improving the knowledge and skills of frontline nurses, we can improve maternal and neonatal outcomes. We also, recommend continuing medical educations (CMEs) within the department and supportive supervision to empower nurse's knowledge on obstetric emergencies.

## Abbreviations

TDHS: : Tanzanian Demographic and Health Survey
PEE: : Pre-eclampsia and eclampsia
MgSO4: : Magnesium Sulphate
WHO: : World Health Organization.
Jhpiego: : Johns Hopkins Program for International Education in Gynaecology and Obstetrics.
